# Multivariate data analysis of growth medium trends affecting antibody glycosylation

**DOI:** 10.1002/btpr.2903

**Published:** 2019-10-18

**Authors:** David N. Powers, Nicholas Trunfio, Sai R. Velugula‐Yellela, Phillip Angart, Anneliese Faustino, Cyrus Agarabi

**Affiliations:** ^1^ U.S. Food and Drug Administration Center for Drug Evaluation and Research, Office of Product Quality, Office of Biotechnology Products, Division of Biotechnology Review and Research II Silver Spring Maryland; ^2^ Sartorius Stedim North America Inc Corporate Research Bohemia NY

**Keywords:** bioprocessing, galactosylation, glutamine, glycosylation, MVDA

## Abstract

Use of multivariate data analysis for the manufacturing of biologics has been increasing due to more widespread use of data‐generating process analytical technologies (PAT) promoted by the US FDA. To generate a large dataset on which to apply these principles, we used an in‐house model CHO DG44 cell line cultured in automated micro bioreactors alongside PAT with four commercial growth media focusing on antibody quality through *N*‐glycosylation profiles. Using univariate analyses, we determined that different media resulted in diverse amounts of terminal galactosylation, high mannose glycoforms, and aglycosylation. Due to the amount of in‐process data generated by PAT instrumentation, multivariate data analysis was necessary to ascertain which variables best modeled our glycan profile findings. Our principal component analysis revealed components that represent the development of glycoforms into terminally galacotosylated forms (G1F and G2F), and another that encompasses maturation out of high mannose glycoforms. The partial least squares model additionally incorporated metabolic values to link these processes to glycan outcomes, especially involving the consumption of glutamine. Overall, these approaches indicated a tradeoff between cellular productivity and product quality in terms of the glycosylation. This work illustrates the use of multivariate analytical approaches that can be applied to complex bioprocessing problems for identifying potential solutions.

## 1 INTRODUCTION

Generation of biological drug products from bioreactors is a complex procedure due to using living cells as the site of manufacturing. Much is still unknown about the relevant cellular processes, so understanding how different variables in bioprocessing affect critical quality attributes (CQA) such as glycosylation and monoclonal antibody (mAb) product titer is of great interest to the biopharmaceutical industry.^1^, ^2^ To study how these variables could potentially contribute to changes in cellular productivity and the CQAs of the product molecule, multivariate approaches are needed to analyze the large amount of data required.^3^ This use of multivariate data analysis (MVDA) in associating cell culture process and material variables to CQAs is sometimes referred to as “fermentanomics.”^4^ MVDA is necessary due to the difficulty in detecting the subtle, yet important, relationships through univariate means in addition to the problems inherent to large datasets such as varying degrees of experimental error, multicollinearity, and missing data.^5^

*N*‐glycosylation is an example of one CQA present in therapeutic mAbs that has large consequences on the efficacy and stability of the protein.^6^ In the mAb immunoglobulin G (IgG), *N*‐glycosylation is found at Asn^297^ in the crystallizable fragment (Fc) of the heavy chain.^7^ This modification takes place in the endoplasmic reticulum (ER) and Golgi apparatus, where a 14‐sugar precursor Glc3Man9GlcNAc2‐ is attached and modified as the protein traverses the Golgi.^8^ The modification process typically involves the loss of the mannose saccharides and replacement with *N*‐acetylglucosamine (GlcNAc) and galactose. Cellular stresses, such as nutrient depletion, can interrupt these enzymatic processes since it affects the available pool of substrate molecules such as nucleotide sugars.^9^, ^10^ The altered rates at which the enzymes modify the polysaccharide chains can result in the different glycoforms, which collectively form the glycan profile of the protein.

These alterations of the cellular environment that impact the mAb glycan profile can have a profound impact on the resulting drug's quality. As the activity of a mAb drug is commonly mediated through antibody‐dependent cellular cytotoxicity (ADCC) or complement‐dependent cytotoxicity, the antibody glycoform can affect these processes by either facilitating or hindering recruitment of necessary interactors. For example, glycans that feature a core fucose will reduce ADCC activity due to the moiety interfering with Fcγ receptor interaction.^11^ The glycosylation state of the protein can additionally affect stability, immunogenicity, and clearance rate.^12^, ^13^ Due to the importance of *N*‐glycosylation in drug quality, further understanding of the variables and processes that affect its outcome is warranted. In this vein, supplementation strategies where the additions were comprised of sugars, metals and amino acids have been shown to directly affect the produced glycan profile.^14^, ^15^ Aglycosylation, where the protein lacks a glycan residue at the Asn^297^ site, is another possible outcome. Nutrient depletion, such as glucose, has been shown to result in this change.^16^ Due to the role of the glycan modification in protein binding, its absence has a marked effect on the protein properties: protein aggregation, reduced stability, and altered pharmacokinetic properties.^17^, ^18^, ^19^ A wide variety of glycan outcomes are possible and there are many bioprocessing and culture medium variables that can affect this process, necessitating the need for multivariate analysis.

To generate a dataset with an adequate number of replicates given the large number of potential inputs involved, we used the ambr®15 automated micro bioreactor system (Sartorius, Hartfordshire, UK). This automated, parallel cell culture platform allows a suitable dataset for multivariate analysis to be generated because many cell culture experiments can be simultaneously run with minimal spurious batch‐to‐batch variability caused by differences in seeding density, cell culture operation, environmental conditions, and media preparation. An in‐house model IgG_1_ producing CHO DG44 cell line was used for these cultures, with the system run in batch mode to accommodate for the vessels' small sizes which could not sustain a reasonable sampling frequency over a longer fed‐batch culture while maintaining the minimum reactor volume necessary for operation. Earlier studies were used to determine our selection of media: Ex‐Cell Advanced (SAFC), CD OptiCHO (Thermo Fisher), PowerCHO2 (Lonza), ProCHO5 (Lonza).^20^ Due to the small size of the micro bioreactors (15 ml, with a minimum volume of 10 ml), the breadth of in‐process analytics that could be performed, such as technical replicates of in‐process measurements, was limited. As such, glycosylation was the only product quality attribute evaluated on the final harvested product.

Due to the quantity of data collected and the difficulty of identifying relationships in complex datasets with only univariate analyses, we used two multivariate analysis techniques to uncover these interactions: principal component analysis (PCA) and partial least squares regressions (PLS). This is possible because the measured variables, collectively referred to as the feature space, vary collinearly with one another; for example, an increase in the abundance (percentage) of one glycoform must result in an equal cumulative decrease in the abundance of the remaining glycoforms. PCA exploits this collinearity by projecting the feature space onto a set of latent variables, called principal components, that describe the orthogonal variations in the original feature space.^21^ Collectively, latent variable data is referred to as the score space, as observations on these new principal components are called scores. Each observation will have a score value associated with it for each of the principal components. Observations that are similar across many of the original variables will appear clustered together in the projection. Therefore, any observations that deviate from the others can be seen. PLS was used to find the functional relationship between bioreactor process parameters and glycan outcomes, such as high mannose (HM) glycoforms and altered proportions of terminal galactosylation.

## 2 RESULTS

**Figure 1:**
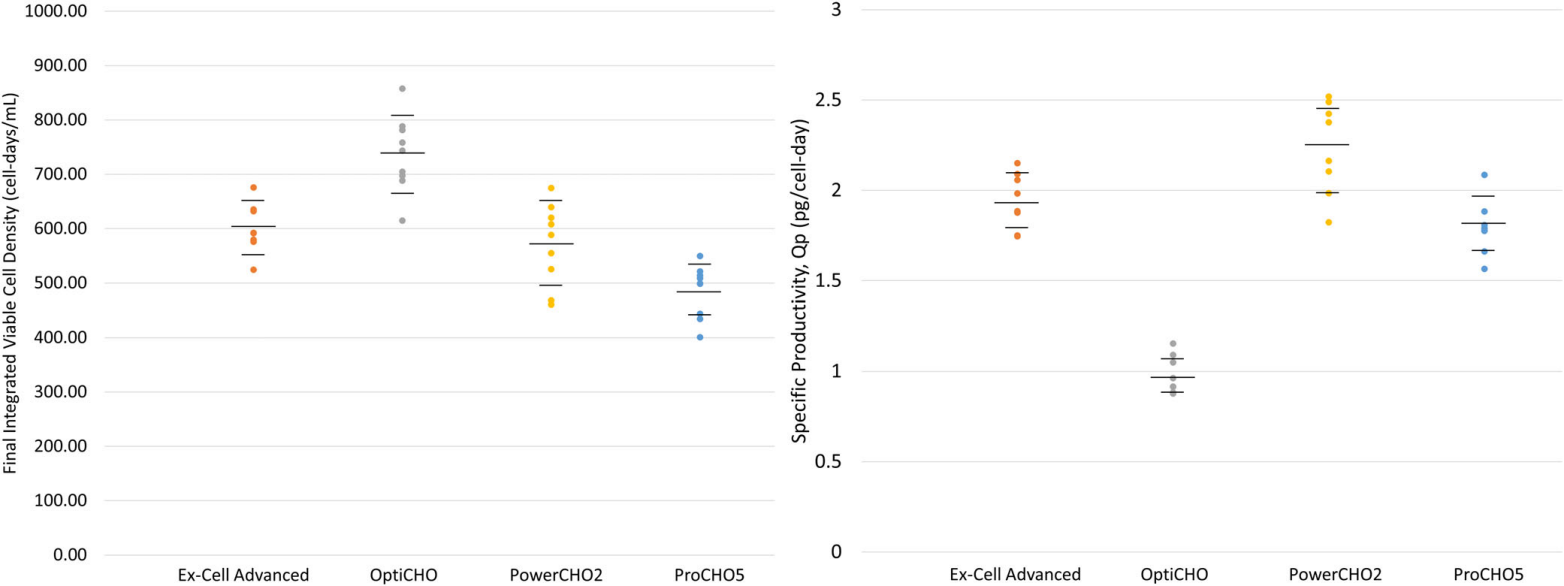
Representative bioreactor growth profiles sorted by media Technical dot plots depict the final integrated viable cell density (IVCD) and specific productivity across all culture conditions for Ex‐Cell Advanced, OptiCHO, PowerCHO2, and ProCHO5 media

We sought to generate a complex dataset from a model IgG_1_ antibody producing bioprocess for use in MVDA using the following commercially available media: Ex‐Cell Advanced, OptiCHO, PowerCHO2, and ProCHO5. We used our in‐house CHO DG44 cell line at an inoculation density of ~1 × 10^6^ cells/ml. Nine micro bioreactors were prepared with each of the four media. One of the bioreactors containing Ex‐Cell Advanced was lost, resulting in a total of 35 bioreactors successfully run. The bioreactors were operated within normal ranges found in bioprocessing with minor differences in the levels of nutrients supplemented. The micro bioreactors were run for 8 days, after which the mAb was collected and purified. Figure 1 displays technical dot plots with the final integrated viable cell density (IVCD) and specific productivity profiles categorized by culture medium, where each dot represents an individual micro bioreactor culture. The IVCD is a calculated variable that represents the cumulative viable cell density over the course of the whole bioreactor run, measured in cell‐days/ml (further details for this variable, as well as the following metabolite values, can be found in Section 4.9). Cultures grown in Ex‐Cell Advanced and PowerCHO2 have comparable IVCD, while this value is lowest in ProCHO5 and highest in OptiCHO. OptiCHO also featured the lowest specific productivity, which was roughly the same in the other media.

**Figure 2:**
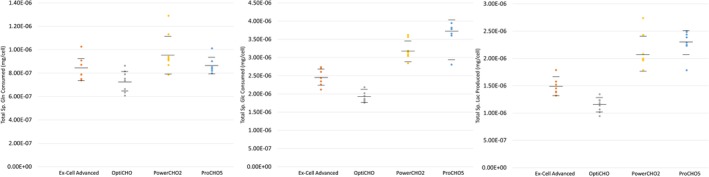
Representative consumption and production within the micro bioreactors sorted by media. Total specific consumption of glutamine (Gln) and glucose (Glu) and lactate (Lac) production across all culture conditions for Ex‐Cell Advanced, OptiCHO, PowerCHO2, and ProCHO5 in terms of total mass per cell (mg/cell)

In‐process measurements for the concentrations of glutamine (Gln), glucose (Glc), and lactate (Lac) were performed using a Bioprofile Flex Analyzer. We used the measured values to calculate the total specific amounts consumed/produced per cell within the micro bioreactors over the total culture life, the resultant technical dot plots of which are shown in Figure 2. Overall, OptiCHO featured the lowest consumption of glutamine and glucose while also producing the lowest amounts of lactate. Likewise, Ex‐Cell Advanced had cultures that consumed/produced in the middle, while the highest consumers/producers were PowerCHO2 and ProCHO5 containing micro bioreactors.

Our next step was to assess if any of the differences we observed in the cell growth and nutrient profiles affected the glycosylation state of the IgG_1_ antibody that was produced. Due to the limited bioreactor volume size, the product mAb was only harvested at the end of the run; because the mAb accumulates in the vessel over time, the final harvest material is representative of the average mAb produced throughout the varied process conditions of the cell culture. Additionally, the average mAb will more closely resemble protein produced at the end of the cell culture due to more protein being produced at the higher cell densities that are reached in the latter stages of the process, and due to the cells' increased productivity as they enter the stationary phase during this time. The purified antibody was analyzed for its glycan profile and heavy chain size variants using mass spectrometry and capillary electrophoresis, respectively. The combined results for these analyses are shown in Table 1. The data in Table 1 is categorized by culture medium and analytical technique, as fluorescence mass spectrometry was used to quantify the numerical percentages of all the glycoforms, while reduced capillary electrophoresis (rCE‐SDS) was used to compute the amount of aglycosylation antibody heavy chains. The results of all biological and three technical replicates each were mean averaged to obtain the values shown in Table 1; the values that are highest for each glycan type are bolded and underlined. Based on the glycan species we observed in our analysis, we used the following groupings: G0F, G1F, G2F, HM (this category contains Mannose 4 to Mannose 9 which consist of mannose oligosaccharide clusters bound to the 2 GlcNAc core), and Other (this category consists of the uncommon glycoforms such as G0F‐N and nonfucosylated glycoforms like G0, G1, and G2). The “Other” category contained less than 5% of the overall glycans present. These groups of glycan species, HM and other, were created to help in data visualization and to simplify the data used to detect significant trends. The sum of terminal galactosylated species (G1F and G2F as opposed to G0F which features no galactosylation) varied greatly based on media: ProCHO5 cultures produced the most G1F and G2F of all the media tested, while OptiCHO produced the most G0F (more than G1F and G2F combined, 53.189% vs. 32.423%). These values could indicate that ProCHO5 medium promotes trafficking through the Golgi apparatus and/or galactosyltransferase enzymatic activity responsible for galactosylation more efficiently than in other media.^22^

**Table 1:** Glycosylation profiles by growth medium

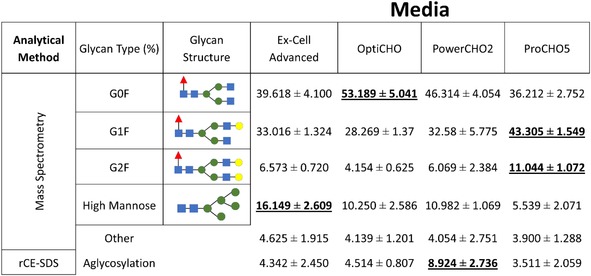

*Note*: Glycosylation profiles by medium. The numbers represent the percentage of the total glycan profile for all labeled glycan data obtained by mass spectrometry which sum to 100%. The rCE‐SDS aglycosylation values represent the percentage of antibody heavy chains are not glycosylated versus those that are. The error values indicated are standard deviation values for all biological replicates and technical replicates measured. The bold values are the highest values for each glycan type.

Culture medium is also associated with the abundance of HM glycoforms which can be characteristic of cellular stress and causes incomplete processing during *N*‐glycan biosynthesis.^6^, ^23^, ^24^ This cellular stress can manifest differently, either as a reduction in galactosylation, or increases in either HM glycoforms or aglycosylation. For example, Ex‐Cell Advanced cultures displayed the largest amounts of HM glycoforms, while PowerCHO2 had the most aglycosylation.

Table 1 shows that culture medium significantly affects the total product glycan profile. Nutrient related cellular stress can cause both an increase of immature glycoforms such as HM and an increase in antibody aglycosylation, but the conditions that result in production of aglycosylated mAbs are not well characterized and do not appear to overlap with those that result in immature glycoforms.^16^, ^22^ As mentioned earlier, glucose depletion has been shown to be responsible for aglycosylation outcomes, though we did not find any evidence of glucose deficiency in the PowerCHO2 vessels (Figure 2). We note that the conditions which result in aglycosylation do not appear to be correlated with those that result in low terminal galacosylation or increased HM species. Our work illustrates the need for fully characterizing the in‐process parameters that result in altered glycosylation states that will modify the therapeutic properties of the antibody, especially when the drug mechanism requires Fc‐binding ligands.

Due to the complex interplay between the different cellular functions affecting growth, metabolism, and glycan outcomes, we reasoned that data driven multivariate analysis would be required to understand the correlation structure that relates the harvested mAbs' quality and the in‐process variables. Accordingly, the exact relationship between media selection and product quality is obscured for multiple reasons. First, the identities of the chemicals contained within commercial growth media are proprietary and unknown. Second, the relationship between the growth measurements, metabolite measurements and final product quality is governed by metabolic pathways containing complex reaction networks that have not been fully characterized. Due to this, we used MVDA techniques, such as PCA and PLS, to find a set of latent variables that describe the variability seen in the measured data and calculated variables when the exact biological relationship between model features is unknown. We only used the mass spectrometry data for the MVDA since mass spectrometry and rCE‐SDS are vastly different techniques that measure attributes on different analytes.

PCA was performed to assess the suitability of using MVDA to characterize the impact of media selection on antibody glycosylation and productivity. The model's features, **X**, are comprised of only the titer and glycosylation profiles from each of the 35 micro bioreactors; the culture medium and other in‐process variables were not included in this initial model, which is summarized in Table 2. Sevenfold cross‐validation was used to determine *Q* ^2^ from predicted values of the excluded data. In order to prevent overfitting, the number of extracted principal components was selected to maximize *Q* ^2^; thus, the predictive power of the model was also maximized. More than 90% of the variabilities in the titer and glycosylation profiles are characterized by the two extracted principal components, while the high *Q* ^2^ value signifies that the results of the model have high predictive power and are not based on spurious correlations.

**Table 2:** Summary statistics for the profile models

Model	A	*R* ^2^(X)	*R* ^2^(Y)	*Q* ^2^
PCA	2	0.903	‐	0.605
PLS	3	0.810	0.729	0.586

*Note*: Model summary statistics for the PCA model characterizing the impact of media selection on antibody glycosylation and for the PLS model used to predict the glycan distributions.

**Figure 3:**
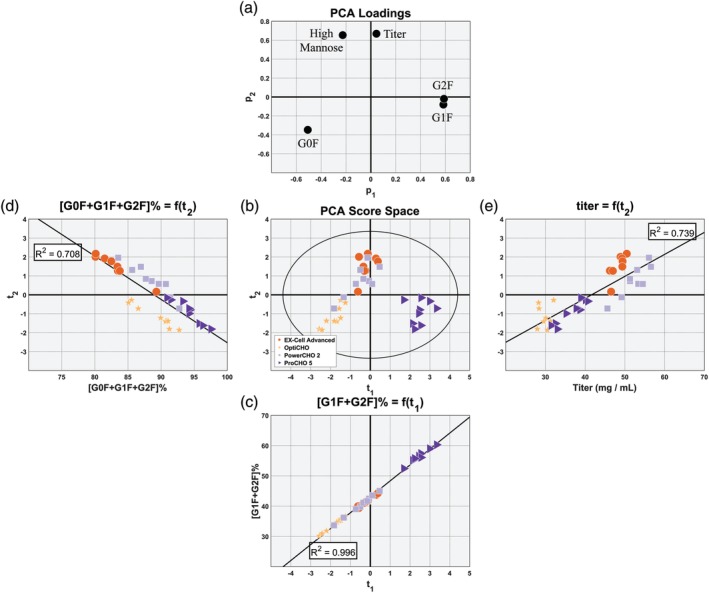
Principal component analysis model to characterize impact of media selection on the glycosylation profile and titer. A and B show the PCA model's loadings and score space. Part C shows a principal component regression (PCR) demonstrating that the first principal component describes the degree to which the cells were able to achieve terminal galactosylation. D and E show that the second principal component characterizes the degree to which the cells can convert high mannose glycoforms to G0F and that the metabolic processes associated elevated conversion are inversely correlated with the metabolic process associated with protein production

The PCA model extracted two principal components suggesting that there are two latent variables which characterize the impact of media selection on titer and product quality; the model's loadings, shown in Figure 3a, can provide insight into how the media selection has such an impact. The first principal component's loadings, **p**_1_, show that the first principal component is inversely correlated with the immature HM and intermediary G0F glycoforms, as depicted by their location in the left side of the loadings plot. **p**_1_ also indicates that the first principal component is positively correlated with the terminally galactosylated G1F and G2F glycoforms, as evidenced by their location in the right side of the loadings plot. Taken together, this suggests that the first principal component is characterizing the correlation structure that relates the impact of media selection on the cells' ability to achieve terminal galactosylation. This is also evident in the model's score space, shown in Figure 3b when considering the first principal component's scores, **t**_1_. Cell cultures grown in OptiCHO are the least efficient at achieving terminal galactosylation, having the lowest sum of G1F and G2F species, and their projections appear furthest to the left in the score space. Moving to the right, it can be observed that Ex‐Cell Advanced and PowerCHO2, which are moderately efficient at creating the terminally galactosylated species, appear next along **t**_1_ near the central axis. Furthermore, it can be seen that cells grown in ProCHO5, which are the most efficient at achieving terminal galactosylation, appear furthest to the right by a significant margin; this is in good agreement with the univariate analysis derived from Table 1. Further evidence that the first principal component is correlated with the cells ability to achieve terminal galactosylation is provided in Figure 3C where the principal component regression (PCR) expressing the sum of terminally galactosylated species, [G1F + G2F] (%), as a linear function of the first principal component's scores, **t**_1_, is able to capture almost all of the variability present in the amount of terminally galactosylated species (*R* ^2^ = 0.996). Interestingly, the proximity of the loading for titer to the origin suggests that the cells' ability to achieve terminally galactosylated glycoforms is independent of how productive the cells are.

The second principal component's loadings, **p**_2_, show that the second principal component is inversely correlated with the intermediary G0F glycoform, as illustrated by its location in the bottom half of the loadings plot. **p**_2_ also indicates that the second principal component is positively correlated with the immature HM glycoforms and titer, as portrayed by their location in the top half of the loadings plot. Taken together, this suggests that the second principal component is characterizing the correlation structure that relates the impact of media selection on the cells' ability to convert the immature HM glycoforms into the intermediary G0F glycoform. Cell cultures with lower projected scores on the second principal component, **t**_2_, will be characterized by their small proportion of the HM glycoforms and relatively high proportion of the other glycosylation species; the inverse would be true of cell cultures whose projected scores, **t**_2_, are large. This is consistent with the pattern observed in the score space: the cells grown in ProCHO5 and OptiCHO have the smallest amount of HM glycoforms and their projections appear in the bottom half of the score space in Figure 3b; cells grown in PowerCHO2 and Ex‐Cell Advanced have the largest amount of HM glycoforms and their projects appear, largely, in the top half of the score space. This is also consistent with the univariate data in Table 1. Further evidence that the second principal component is correlated with the cells ability to convert HM glycoforms into the G0F glycoform is provided in Figure 3D where the PCR expressing the total amount of all G0F, G1F, and G2F glycoforms, [G0F + G1F + G2F] (%), as a linear function of the second principal component's scores, **t**_2_, is able to describe a majority of the variability in the amount of intermediate and mature glycoforms (*R* ^2^ = 0.708).

In addition, the second principal component's loadings, **p**_2_, suggest that the underlying metabolic phenomena responsible for converting the HM glycoforms into the G0F glycoform are inversely correlated with the metabolic phenomena responsible for increased protein production and that optimizing for titer could have deleterious effects on product quality, and vice versa. Further evidence for this can be seen in Figure 3e; it shows that the PCR that expresses titer as a linear function of **t**_2_ is also able to describe a majority of the variability in titer (*R* ^2^ = 0.739). The fact that the slope of the regression in 3D is negative and the slope of the regression in 3E is positive is further evidence of the inverse relationship between cell productivity and efficiency in converting HM glycoforms to the G0F glycoform.

Having established a set of latent variables that can be used to discriminate between the productivity and glycosylation profiles resulting from cells grown in different media formulations, our next step was to determine if there were metabolic differences in the various cell cultures that were correlated with the observed titers and glycosylation profiles. To accomplish this, a PLS model was created where the response features, **Y**, were comprised of the titer and glycosylation profiles and the regressor features, **X**, contained 32 variables related to growth, metabolite uptake, and metabolite secretion. Specifically, the regressor features were: growth rate (Day 5), IVCD_24_ (Days 6 and 7), specific glutamine uptake rate (Days 0, 1, 2, 3, 4, and 5), specific glucose uptake rate (Days 1, 2, 3, and 6), specific lactate secretion rates (Days 5 and 6), specific glutamine uptake_24_ (Days 1, 2, 3, 4, 5, and 6), cumulative specific glutamine uptake (Days 1, 2, and 3), specific glucose uptake_24_ (Days 3, 4, and 6), cumulative specific glucose uptake (Days 3, 4, 5, and 6) and specific lactate production_24_ (Day 6). Table 2 contains the model summary statistics: the *R* ^2^(X) and *R* ^2^(Y) values indicate that over 80% of the process measurement variabilities and over 70% of the titer and glycosylation profiles variabilities, respectively, are embodied by the three principal components extracted by the model. It can be seen that despite extracting more principal components the PLS model is able to describe less of the titer and glycosylation variabilities than is described by the PCA model, as evidenced by the PLS model's *R* ^2^(Y) value being lower than the PCA model's *R* ^2^ value. This is a necessary consequence of trying to find the correlation structure in the process measurements that are related to the changes in titer and glycosylation. Not all process measurements that affect titer and glyocsylation have been measured; therefore, only the titer and glycosylation variabilities that are correlated with the process measurements should be characterized by the PLS model. The high *Q* ^2^ value, relative to the *R* ^2^ values, indicates that the PLS model has good predictive power and is not based on spurious correlations.

**Figure 4:**
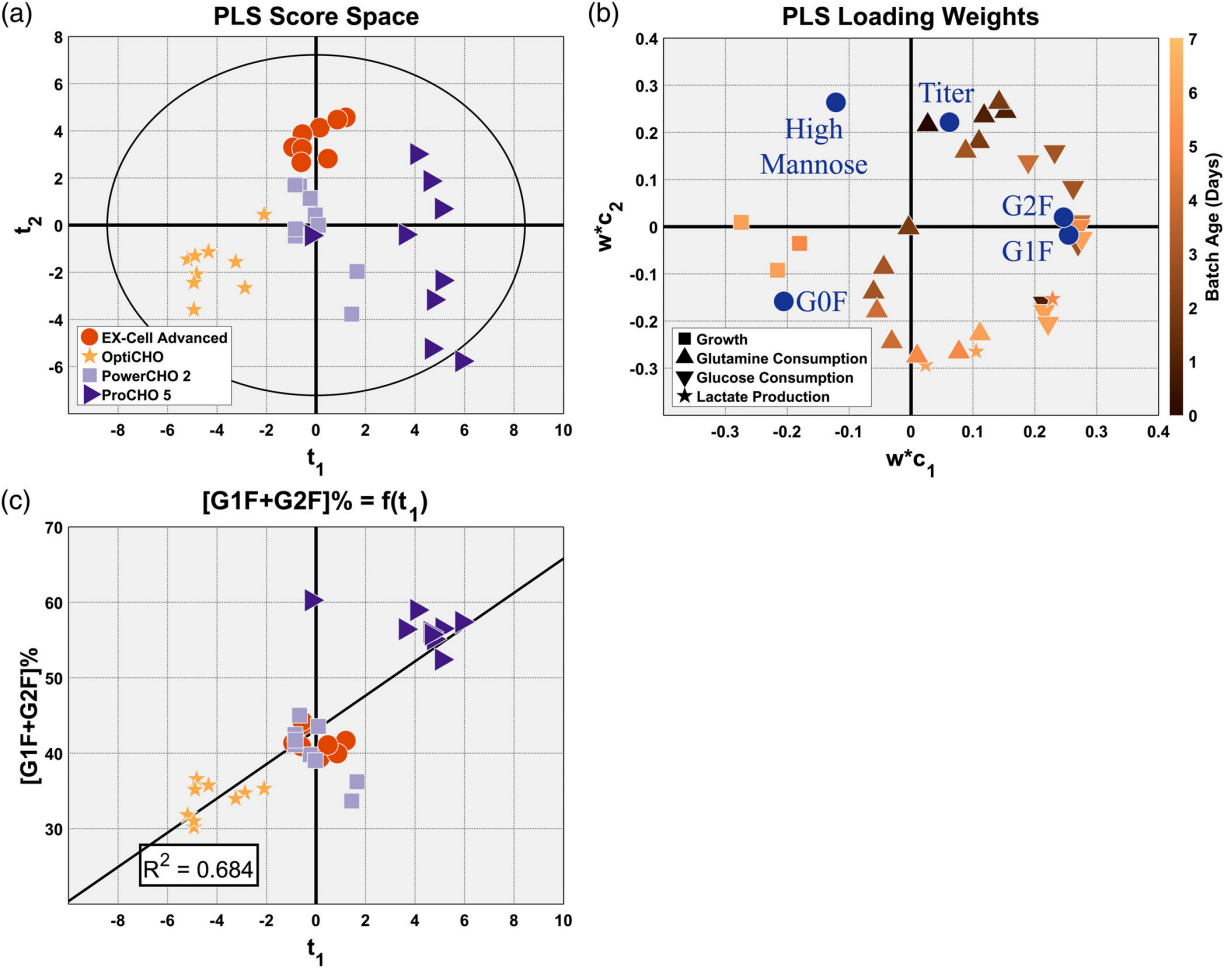
Partial least squares (PLS) regression model to predict the glycosylation profile and titer––Principal Components 1 and 2. A shows PLS model's score space. B shows a principal component regression demonstrating the extent to which the first principal component describes the degree cells were able to achieve terminal galactosylation. Part C shows the PLS model's loading weights

Figure 4a,c shows the score space and loading weights plots, respectively, for the PLS model. The **Y** block features' loading weights, shown as blue circles in Figure 4c, have a very similar pattern to the one observed in the PCA model's loadings depicted in Figure 3a. This indicates that the first two principal components extracted by the PLS model can be interpreted as characterizing the same phenomena as those extracted by the PCA model: the first principal component is positively correlated with the cells' ability to achieve terminally galactosylated glycoforms and the second principal component is positively correlated with titer and inversely correlated with the cells' ability to convert the immature HM glycoforms into the intermediary G0F glycoform. However, even though the pattern of the **Y** block features' loading weights in Figure 4c is similar to the pattern of the loadings in Figure 3a, there are some small differences that could affect the relevance of the PCA model's interpretation for the PLS model. This is most evident by comparing the PLS model's score space, Figure 4a, with the PCA model's score space Figure 3b. There are still clusters for each of the four media types, and the clusters are located in similar regions of the score space for both models; however, the distributions of these clusters are substantially different, especially along the second principal component.

The PCA model's interpretation is most relevant for the PLS model when considering only the PLS model's first principal component. Moving across the score space in Figure 4a from left‐to‐right, OptiCHO has the lowest amount of terminal galactosylation, followed by Ex‐Cell Advanced and PowerCHO2 having an intermediate amount of terminal galactosylation and ProCHO5 having the most terminal galactosylation by a large margin. Further evidence that the latent variables from both models are correlated with the same underlying biological phenomena can be seen by performing a PCR that expresses [G1F + G2F] (%) as a linear function of the PLS model's first principal component's scores, **t**_1_, shown in Figure 4b, and comparing it with the PCR for the PCA model, shown in Figure 3c. The PLS model captured less of the terminal galactosylation variability than the PCA model, *R* ^2^ = 0.684 versus *R* ^2^ = 0.966, respectively. This implies that up to two‐thirds of the terminal galactosylation variabilities observed are correlated with the growth and metabolite measurements used as regressors in the **X** block. It also implies that at least one‐third of the differences in terminal galactosylation observed cannot be attributed to factors considered in this work. The distribution of the observations on the PCR in Figure 4b suggests that the measured process parameters are correlated with the large changes in terminal galactosylation seen between media but that they are not correlated with the subtler terminal galactosylation variabilities seen within each media. Therefore, the model is well‐suited for establishing the correlation structure between terminal galactosylation and the growth/metabolite utilization that occurs in cells grown in the different media conditions.

The relationship between the overall glycosylation enzymatic pathway efficiency and cellular metabolism can be interpreted from the loadings weights for the first principal component (w*c_1_) in Figure 4c. In order to make the loading weights more interpretable all parameters related to glucose are plotted with a single type of marker (upside down triangle), and the same was done for the parameters related to growth, glutamine (triangle) and lactate (star) as well. Darker marker coloration indicates earlier in batch age, while light coloration signifies the end of the batch. The binary relationship between each response and regressor can be found in the model's coefficients given in Figure S1. As the overall terminal galactosylation efficiency is positively correlated with the first principal component, the in‐process variables whose loading weights fall in the left‐half of Figure 4c are inversely correlated with the cells ability to achieve terminal galactosylation and those variables whose loading weights fall in the right‐half of Figure 4c are positively correlated with the cells ability to achieve terminal galactosylation. Thus, it can be seen that terminal galactosylation efficiency has a strong positive correlation with glucose consumption throughout the batch, a moderate inverse correlation with cell growth at the end of the batch, a moderate positive correlation with lactate production at the end of the batch and an ambiguous relationship with glutamine consumption. In this context, the strength of the correlation is determined by the loading weights proximity to the origin and is only meant to imply strength relative to one another for this principal component.

These results imply that cells whose metabolic processes utilize more glucose should be more likely to create protein that has achieved terminal galactosylation. It is important to note that because a design of experiments (DoE) was not performed to independently set **X** block measurements, a causal relationship between glucose utilization and glycosylation efficiency was not established; we are claiming that differences in media composition impact aspects of cellular metabolism responsible for glucose utilization and aspects of cellular metabolism responsible for achieving terminal galactosylation and that there appears to be a positive correlation between these two processes. The loading weights at the end of the culture, Days 5, 6, and 7, provide more detail to the interpretation; they imply that lactate production is positively correlated with terminal galactosylation efficiency and growth is inversely correlated with terminal galactosylation efficiency. Together, we interpret this as suggesting that cells achieving a high degree of terminal galactosylation are utilizing the increased amount of glucose as an energy source at the end of the culture, as evidenced by the positive correlation with lactate production, but this energy is being used by metabolic processes unrelated to growth, as evidenced by the inverse correlation with cell growth.

**Figure 5:**
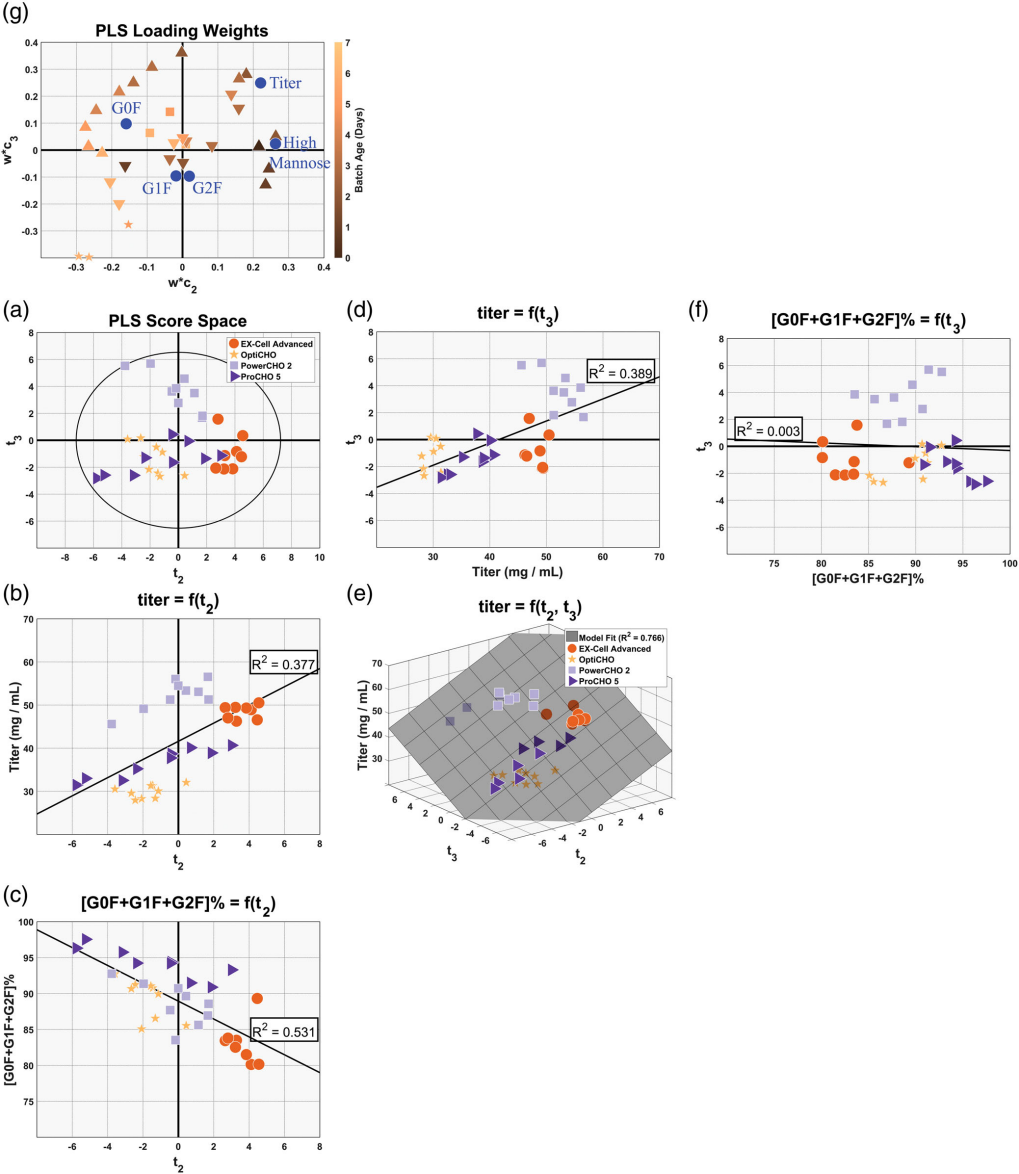
Partial least squares (PLS) regression model to predict the glycosylation profile and titer––Principal Components 2 and 3. A shows the PLS model's score space. B and C show principal component regressions (PCR) demonstrating that the second principal component describes the tradeoff between productivity and the cells ability to convert immature high mannose glycoforms to the G0F. B, D, and E show PCR demonstrating that both the second and third principal components are needed to characterize a majority of the variability seen in the titer measurements. B, D, and F show PCR demonstrating that roughly half of the variability seen in the titer measurements is independent of the variability seen in the cells ability to convert high mannose glycoforms to G0F. G shows the PLS model's loading weights

Examining the score values for the second principal component, **t**_2_, in Figure 3b for the PCA model and in Figure 4a for the PLS model it is clear that the second principal component does not describe the same phenomena in the PLS model as in the PCA model––at least not in the same way. It will be shown here that the tradeoff between productivity and the conversion of HM species to G0F captured by the PCA model in a single latent variable may be described more completely by two latent variables with one of them being correlated with this tradeoff and the second one that is correlated with the aspects of productivity that are independent of the conversion of HM to G0F. These two phenomena are captured by principal Components 2 and 3 in the PLS model whose score space can be seen in Figure 5a.

It can be seen from Figure 5b that the second principal component is positively correlated with titer from the PCR that expresses titer as a function of **t**_2_. However, the fraction of variability in the titer measurements described by **t**_2_ in the PLS model is roughly half of that described by **t**_2_ in the PCA model (*R* ^2^ = 0.377 vs. *R* ^2^ = 0.739, respectively). The remaining fraction of variability in the titer measurement is described by the third principal component, as shown by the PCR in Figure 5d that expresses titer as a linear function of **t**_3_ (*R* ^2^ = 0.389). Furthermore, a multiple linear regression that expresses titer as a function of **t**_2_ and **t**_3_ captures the majority of the variability seen in the titer measurements (*R* ^2^ = 0.766), as seen in Figure 5e; this is comparable to the fraction of variability captured by a single component in the original PCA model (*R* ^2^ = 0.739).

It can be seen from Figure 5c that the second principal component is also inversely correlated with the cells ability to convert HM glycoforms to G0F from PCR that expresses [G0F + G1F + G2F] (%) as a function of **t**_2_ (*R* ^2^ = 0.531). Unlike titer, a second PCR (Figure 5f) that expresses [G0F + G1F + G2F] (%) as a function of **t**_3_ indicates that the cells ability to convert HM glycoforms to G0F is not correlated with the latent variable extracted into the third principal component (*R* ^2^ = 0.003). Together, this implies that only the second principal component represents the tradeoff between productivity and converting HM to G0F. The high *R* ^2^ value for the PLS model relative to the PCA model, *R* ^2^ = 0.531 versus *R* ^2^ = 0.708, respectively, indicates that 15% or more of the variability in the cells ability to convert HM glycoforms to G0F that was captured by the PCA model is not related to the growth and metabolite factors considered here.

Similar to the analysis of loadings from the PCA model, the loading weights from the PLS model, shown in Figure 5g, can provide insight into how the growth and metabolite measurements relate to the productivity and product quality outcomes. The second principal components' loading weights, w*c_2_, can be analyzed by considering those loading weights that fall in the right‐half of Figure 5g as being positively correlated with titer and inversely correlated with the cells' ability to convert immature HM glycoforms to G0F; the opposite is also true for those loading weights that fall in the left‐half of the plot. Therefore, it can be seen that cell cultures with elevated glutamine consumption during the lag and early exponential growth phases (Days 0–4), but lowered glutamine consumption during the stationary phase (Days 5 and 6) tended to result in both more protein, and protein that contained a higher proportion of immature HM glycoforms. For analogous reasons, the loading weights suggest that cultures consuming lowered levels of glucose and producing lowered levels of lactate during the stationary phase (Days 5 and 6) tended to result in more protein which contained a higher proportion of immature glycoforms. Similarly, an analysis of the loading weights for principal Component 3, w*c_3_, suggest that cultures with elevated glutamine consumption during the exponential growth phase (Days 2–5) tend to produce more protein and cultures with lowered levels of glucose consumption and lactate production during the stationary phase (Days 5 and 6) tended to result in more protein as well.

Taken together, these results suggest that cells that utilize more glucose for energy during the stationary phase of the culture will also result in cells with lower productivity. It should be noted again that we are not suggesting the aspects of metabolism related to consuming glucose for energy have a causal relationship with the aspects of metabolism related to protein production; rather we are suggesting that media selection has an impact on both of these aspects of cellular metabolism and that there appears to be an inverse correlation between these two processes. In addition, the results indicate that cells that consume more glutamine during the lag and exponential phases tend to produce more protein. The elevated consumption of glutamine during the lag phase appears to come at a cost: the cells that consume elevated levels of glutamine during the lag phase also tend to consume less glutamine during the stationary phase and produce protein that has a lower degree of G0F, G1F, and G2F glycoforms. However, elevated levels of glutamine consumption during the exponential growth phase does not appear to suffer from this drawback, as cells with elevated glutamine consumption during the exponential growth phase tended to produce more protein without having an effect on the cells' ability to convert HM glycoforms to G0F.

## 3 DISCUSSION

Multivariate analysis of our complex bioprocessing dataset allowed us to identify biologically relevant relationships that would not be found using only univariate analyses on the individual factors. Initially, we were able to show that the cells grown in different media produced protein with different glycan profiles, as each medium appeared to promote a separate type of glycan species. For example, Ex‐Cell Advanced had the highest amount of HM glycoforms and PowerCHO2 featured the most aglycosylation, while ProCHO5 resulted in the largest degree of terminal galactosylation. MVDA was needed to identify the in‐process variables that were correlated with the glycan observations. We established that factors linked to cell growth and glucose consumption were correlated with overall terminal galactosylation efficiency. Specifically, cell cultures that consumed more glucose throughout the batch and grew more slowly during the stationary phase tended to have lower amounts of HM outcomes and increased terminal galactosylation. Additionally, glutamine consumption was found to be related to the location of enzymatic bottlenecks in the glycosylation reaction network and overall productivity. Specifically, cell cultures that consumed an abundance of glutamine during the lag phase tended to produce more protein, but that protein tended to have more immature HM glycoforms. Furthermore, cell cultures that consumed elevated levels of glutamine during the exponential growth phase tended to produce more protein without any change in the glycosylation profile. Lastly, cell cultures that consumed elevated levels of glutamine during the stationary phase tended to produce less protein, but this product contained less of the immature HM glycoforms. It is important to note that because the in‐process variables could not be set independently of one another that we are not concluding that the relationships found are causal; rather, the data indicates that the same underlying metabolic conditions responsible for productivity and glycosylation outcomes are also responsible for the differences seen in cell growth and metabolite utilization.

Glutamine is a commonly measured nutrient in bioreactors since it supports cells with high energy demands (as an alternative energy source) and that synthesize large amounts of proteins. However, glutamine is unstable when not in a dipeptide form and breaks down into ammonia and pyroglutamate.^25^ The resulting increase in ammonia that results from excess glutamine can cause an elevation in pH which decreases the functionality of the glycosyltransferases, such as those responsible for galactosylation. While the pH in the bioreactors is carefully controlled, there might still be small increases in the intracellular pH that can result in the decrease of galatosylated glycoforms.^26^ Alternatively, as glutamine supplementation causes increases in glucosamine levels it is likely that loss of galactosylation results from this effect as well.^27^ Here, we report that we also find that elevated glutamine consumption during the lag phase can result in a greater abundance of HM glycoforms as well. Collectively, these results show the importance of PAT to measure levels of nutrients such as glutamine to maintain concentrations that will not adversely affect product quality.

MVDA allowed us to validate experimental findings already established through different experimental approaches while also discovering new growth, medium, and productivity related trends. The increasing complexity of bioprocessing and costs associated with running bioreactors necessitates better understanding on how different input variables affect product quality. Future bioreactor studies to verify that metabolite and growth profile factors can cause changes in the HM glycoform rates will be used to validate our current findings. In this study, we uncovered growth medium parameters that were significantly linked to changes in the final antibody product glycan profile, which will be studied further with the overarching goal of tailored CQA control.

## 4 MATERIALS AND METHODS

### 4.1 Cell culture reagents

Four media were used for this study: CD Ex‐Cell Advanced (SAFC, Lenexa, KS), CD OptiCHO (Thermo Fisher Scientific, Waltham, MA), PowerCHO2 (Lonza, Walkersville, MD), and ProCHO5 (Lonza, Walkersville, MD). 8 mM glutamine (Corning, Manassas, VA) and 1X Penicillin/Streptomycin (Corning, Oneonta, NY) were added during seed train. Additional supplements added during inoculation included: Glutamine (200 mM), Essential Amino Acids (50X; EAA; Thermo Fisher Scientific, Grand Island, NY), and Vitamins (100X; Thermo Fisher Scientific, Grand Island, NY).

### 4.2 Cell culture instrumentation and process

This procedure is demonstrated in *Journal of Visualized Experiments*.^28^ Briefly, the ambr®15 system (Sartorius, Hertfordshire, UK) was run in batch mode while using four culture stations to support running 36 micro bioreactors (only sparged). An in‐house CHO DG44 cell line was inoculated at a density of 1 × 10^6^ cells/ml. The same process parameter set points were used for all reactor vessels: agitation rate = 1,000 rpm, dissolved oxygen (DO) = 50%, pH = 7.1 ± 0.05, temperature = 37°C. Due to the small micro bioreactor volume average of 15 ml, only ~2 ml of medium per day were pulled from each micro bioreactor. Because of this, the product antibodies could only be characterized after harvesting on the 8th day. The micro bioreactors were inoculated using standard seed train protocol.^20^ The reactor vessels were charged with medium, inoculated and sampled, with the liquid handler used for additions. The viable cell density (VCD) and viability growth measurements were measured daily using the Vi‐Cell XR cell viability analyzer (Beckman Coulter, Brea, CA). Metabolite measurements for glucose, glutamine and lactate, were measured daily for the first 6 days of the cell culture using a BioProfile Flex Analyzer (Nova Biomedical, Waltham, MA). In‐house CO_2_ and 1 M NaOH (Thermo Fisher Scientific, Fair Lawn, NJ) were used to maintain the pH, where the amount of CO_2_ input is managed by the system. The antibody product was harvested after 8 days of growth. Use of the Octet Red 96 (Pall Life Sciences, Port Washington, NY) for the calculation of specific productivity (Q_p_) has been described previously.^20^

### 4.3 Monoclonal antibody purification

A 0.22 μm PVDF membrane was used to sterile filter harvest cell culture fluid. The purification procedure is demonstrated in *Journal of Visualized Experiments*.^29^

### 4.4 Concentration of purified antibody

This procedure is shown in *Journal of Visualized Experiments*.^29^ After purification, a Thermo Scientific NanoDrop One microvolume UV‐Vis spectrophotometer was used to determine the sample concentration while the protein extinction coefficient of 13.7 at 280 nm for a 1% IgG solution was used in the calculation of the antibody concentration. A solution of 0.1 M acetic acid neutralized to pH 5.5 with Tris Base (Sample Buffer) was used to blank the instrument.

### 4.5 Aglycosylation of antibody heavy chain

Reduced capillary electrophoresis‐sodium dodecyl sulfate method was used to determine the percentage of aglycosylated (nonglycosylated) heavy chain of purified antibody. The size of the aglycosylated heavy chain was tested using PNGase F (Promega, cat#V4483A) and monitoring peak shift from glycosylated to aglycosylated heavy chain with and without PNGase F treatment (data not shown). The GXII HT Touch micro capillary electrophoresis system (Perkin Elmer) was used with a Protein Express Assay LabChip (PerkinElmer, cat#760499) and associated Protein Clear HR Reagent Kit. The LabChip and sample preparation were performed as written in the official protocol. Briefly, Protein A purified samples were diluted to 1 mg/ml in storage buffer by mixing 2.5 μl of sample with 35 mM dithiothreitol (DTT) containing sample buffer. The samples were denatured at 70°C for 10 min and then 35 μl of water were added. VeriMAb Standard was used for system calibration and samples were analyzed in triplicate (technical replicates). Peak integration for exported electropherograms were exported to Empower 3 FR2 where the Gaussian skim and shoulder detect features were utilized.

### 4.6 Glycan characterization

*Rapi*Flour‐MS Dextran Calibration Ladder was used to determine glycan identities with the mass information used to validate identifications. Labeled glycan samples were run in three technical replicates. For more information refer to the *Journal of Visualized Experiments*.^29^

### 4.7 Software

MATLAB R2017 (Mathworks, Natick, MA) was used for data preprocessing, all calculations necessary to estimate the cell culture and cell state parameters, and to generate plots to visualize results. All multivariate data analyses were conducted in SIMCA 14.1 (Umetrics, Sweden).

### 4.8 Cell culture data preprocessing

To overcome time lag effects due to the measurement times, it was necessary to fit a smooth function to each batches' set of observed metabolite and growth measurements. This was accomplished using the Shape Language Modeling toolbox to fit a smooth function to each of the measured time‐series using a cubic interpolating spline with six knots.^30^ In addition to finding the best profile, as defined by least squares, over‐fitting was prevented by using growth, nutrient and metabolic byproduct heuristics as linear constraints in the objective function that minimizes the sum of square error––these constraints are shown in Table 3. The exact locations of the maxima and inflection points were found using an iterative approach with a 0.25‐day grid for each time‐series individually. Sevenfold cross‐validation was used to determine the locations that resulted in the best fitting spline.^31^

**Table 3:** Constraints on interpolating spline for each time‐series

Parameter	Constraints (description)	Constraints (formula)
Glucose (Glc)	Glucose is always positive	∀*t*, 0 ≤ Glc
	Glucose always decreases monotonically	$\forall t,\;\frac{d\;\mathrm{Glc}}{\mathrm{dt}}\leq 0$
	Glucose decelerates to an inflection point where it begins to decrease slower and slower	∃tinf∈0,8:dGlc2dt2≤0,∀t≤tinfdGlc2dt2≥0,∀t≥tinf
Glutamine (Gln)	Glutamine is always positive	∀*t*, 0 ≤ Gln
	Glutamine always decreases monotonically	$\forall t,\;\frac{d\;\mathrm{Gln}}{\mathrm{dt}}\leq 0$
	Glutamine decelerates to an inflection point where it begins to decrease slower and slower	∃tinf∈0,8:dGln2dt2≤0,∀t≤tinfdGln2dt2≥0,∀t≥tinf
Lactate (Lac)	Lactate is always positive	∀*t*, 0 ≤ Lac
	Lactate increases monotonically to a maximum and then decreases monotonically	∃tmax∈0,8:dLacdt≥0,∀t≤tmaxdLacdt≤0,∀t≥tmax
	Lactate accelerates to an inflection point before the maximum where it begins to decelerate	∃tinf∈0tmax:dLac2dt2≥0,∀t≤tinfdLac2dt2≤0,∀t≥tinf
Viability (Via)	Viability is always between 0 and 100%	∀*t*, 0 ≤ Via ≤ 100
	Viability always decreases monotonically	$\forall t,\;\frac{d\;\mathrm{Via}}{\mathrm{dt}}\leq 0$
	Viability decreases linearly until an inflection point where it begins to decelerate	∃tinf∈0,8:dVia2dt2=0,∀t≤tinfdVia2dt2≤0,∀t≥tinf
Viable cell density (VCD)	Viable cell density is always positive	∀*t*, 0 ≤ VCD
	Viable cell density increases monotonically to a maximum and then decreases monotonically	∃tmax∈0,8:dVCDdt≥0,∀t≤tmaxdVCDdt≤0,∀t≥tmax
	Viable cell density accelerates until an inflection point before the maximum where it begins to decelerate	∃tinf∈0tmax:dVCD2dt2≥0,∀t≤tinfdVCD2dt2≤0,∀t≥tinf

After determining our smoothing functions, they can be applied to obtain an estimate of what the measured value would have been if the sample had been drawn at a given time. In order to ensure that the measurement estimates were generated at the same times across all measurements and all batches, the reactor vessel inoculation time was used as the starting time, *t* = 0, for each batch. Then, the measurement estimates were generated by evaluating the smooth functions at *t* = 0, 1, 2,…,8 days after the inoculation time. In total, 39 new estimates of the measured parameters were generated for each batch. A set of nine estimated measurements were generated for each of the two growth measurements from inoculation to harvest on the 8th day. A set of seven estimated measurements were generated for each of the three metabolites during the first 6 days of the cell culture; the average of the resulting estimated glucose, glutamine and lactate profiles are shown in Figure 2.

### 4.9 Cell parameter estimation

There is additional information about the cell culture that can be calculated from the measured data to describe variations seen in product quality between batches. This generates an additional 30 calculated variables, per batch, to be investigated as potential model features that describe the observed product quality variations.

The IVCD describes the summed amount of time that cells spent alive, and by extension growing and producing protein, over an arbitrary time interval; the final values of this variable are shown in Figure 1. This generates an additional 18 calculated variables, per batch. One set of nine variables, denoted as IVCD, corresponds to the total cumulative amount of time from inoculation up to that point in time. The remaining set of nine variables corresponds to the total amount of time that all cells spent alive over the past 24 hr; this receives the notation IVCD_24_.

The average cellular rate at which glucose, glutamine, and lactate are being consumed/produced can be determined by taking the first derivative of the three metabolite profiles with respect to IVCD. The average total amount of each metabolite consumed or produced by the cells can then be obtained by integrating the Cell Derivative profiles with respect to time. The resulting 21 calculated variables representing the cumulative total change per cell can be seen in Figure 2. An additional 21 calculated variables were generated in this step corresponding to the change in metabolite per cell over the previous 24‐hr interval in an analogous manner as was done for IVCD_24_.

For all integral calculations, we used the Newton–Cotes integration formula for 5 points, as shown in Equation 1.

(1) $$\int_{a}^{b}f\left(x\right)\mathrm{dx}=\left(b-a\right)\frac{7\;{\left.f\left(x\right)\right|}_{x=a}+32\;{\left.f\left(x\right)\right|}_{x=a+h}+12\;{\left.f\left(x\right)\right|}_{x=a+2h}+32\;{\left.f\left(x\right)\right|}_{x=a+3h}+7\;{\left.f\left(x\right)\right|}_{x=b}}{90}$$

The integration boundaries are *a* and *b*, *f*(*x*)|_*x* = *a*_ is the parameter estimate when the spline is evaluated at *x* = *a* and the step size is defined by $h=\frac{b-a}{5}$.^32^

All derivatives were calculated using a second order Lagrange polynomial that was fit to the data because some independent variables (i.e., IVCD in the cell derivative calculations) exhibit uneven spacing. This is shown in Equation 2:

(2) $$\;\frac{d\;f\left(x\right)}{\mathrm{dx}}=f\left(x_{0}\right)\frac{2x-x_{1}-x_{2}}{\left(x_{0}-x_{1}\right)\left(x_{0}-x_{2}\right)}+f\left(x_{1}\right)\frac{2x-x_{0}-x_{2}}{\left(x_{1}-x_{0}\right)\left(x_{1}-x_{2}\right)}+f\left(x_{2}\right)\frac{2x-x_{0}-x_{1}}{\left(x_{2}-x_{0}\right)\left(x_{2}-x_{1}\right)}$$

To evaluate the derivative at time point *x* ɛ (*x*_0_, *x*_2_), three consecutive measurement pairs (*x*_0_, *f*(*x*_0_)), (*x*_1_,  *f*(*x*_1_)), (*x*_2_, *f*(*x*_2_)) are used.^32^ The first time point is designated *x* = *x*_0_, the internal data points *x* = *x*_1_ and the final time point *x* = *x*_2_.

### 4.10 Multivariate data analysis

PCA was used to uncover variations in glycan profiles for the antibodies produced by cells cultured in different media that are overlooked in univariate analysis. For each principal component there will be a loading, **p**, for each of the original features; formally, it is defined as the cosine of the angle between the original feature and the new principal component. Collectively, the loading, **p**, describe the relative orientation of the score space with respect to the feature space. This orientation is selected so that the variance of the data's projection into the score space is maximized. The exact relationship between the feature space, **X**, and the score space, **t**, is described by Equation 3. The residual matrix **E** represents the feature space variability not characterized by the number of extracted principal components, A. A is determined by sevenfold cross‐validation to prevent overfitting.^31^ The number of principal components was selected to maximize the model's predictive power, where the fraction of variability in the data excluded from model training that can be predicted by the model, Q,^2^ is used as the metric for assessing predictive power.^23^

(3) $$\;X=\sum_{i=1}^{A}t_{i}p_{i}+E=\mathrm{TP}+E$$

In quantitative regression a set of features, known as regressors, are used to predict another set of features known as responses. Traditional linear least squares regressions are not appropriate for bioprocessing due to the features in each block of data varying collinearly with one another and thus violating the independence assumption in linear least squares regression. Therefore, another multivariate analysis tool, PLS, was used to establish the functional dependence of various glycoforms on in‐process measurements obtained during cell culture and to establish correlations between parameters and glycoforms.

In PLS regression, the response features are projected into the score space according to Equation 4: **u** are the score values of the responses after they have been projected into the score space, **c** are the loadings that relate the responses score space back to the original feature space and **G** is the residual matrix containing the variability in the responses that is not described by the first A principal components.

(4) $$\;Y=\sum_{i=1}^{A}u_{i}c_{i}+G=\mathrm{UC}+G$$

The regressors are projected into the score space according to Equation 3. Unlike in PCA, the loadings, **p**, are not selected to maximize the variance of the scores, **t**. In PLS, the loadings are selected such that the covariance between **u** and **t** is maximized; the latent structures onto which **X** and **Y** are projected are the most relevant for describing the functional relationship between **X** and **Y**. In doing this the **X** scores, **t**, become suitable regressors of **Y** and Equation 4 can be rewritten as seen in Equation 5 where **F** is the model prediction error.

(5) $$\;Y=\sum_{i=1}^{A}t_{i}c_{i}+F=\mathrm{TC}+F$$

Due to the PLS model selecting the loadings in order to maximize the covariance between **u** and **t**, the loadings are not orthogonal and do not describe the independent contribution of each of the **X** block features to the scores. Therefore, the appropriate relationship between the regressor feature space and score space is to use the relationship in Equation 6 where **W** ^*^ is the set of loading weights that estimate the score space **T** from linear combinations of the regressor features, **X**. This can be substituted into Equation 5 to get the final relationship shown in Equation 7; **B** = **W** ^*^ **C** is the matrix of PLS model coefficients that defines the overall relationship between the regressors, **X**, and responses, **Y**.

(6) $$T=XW^{*}$$

(7) $$Y=XW^{*}C+F=\mathrm{XB}+F$$

Similar to PCA, *k*fold cross‐validation^28^ was used to select the optimal number of principal components; A was selected to maximize the model's predictive power, as measured by *Q* ^2^.

### 4.11 Feature selection

In total, there were 150 potential model features: 39 cell culture measurement estimates and 111 additional calculated variables generated from the measurement estimates. Of these potential model features, 9 trivial features were eliminated as they did not vary between batches. For example, the Day 0 lactate measurement is trivial as it is below the limit‐of‐detection. Similarly, the Day 0 value can be excluded for all variables derived by integration because nothing has accumulated when the cell culture is starting.

In order to focus the PLS model's analysis on features relevant for cellular metabolism, only the cell specific metabolite features were used for the analysis and all metabolite concentration data were not used. After this, 79 features remained: they were the time‐series for growth rate, IVCD_24_, IVCD, specific glutamine consumption rate, specific glucose consumption rate, specific lactate production rate, specific glutamine consumed_24_, specific glucose consumed_24_, specific lactate produced_24_, cumulative specific glutamine consumed, cumulative specific glucose consumed, and cumulative specific lactate produced.

A second feature selection step was implemented to keep only those features with the most consistent impact on glycosylation and titer. First, a PLS model was constructed to relate the **X** block, consisting of the 79 variables remaining after the first feature selection step, to the **Y** block comprised of the glycosylation profiles and titer. Then, variable importance in projection (VIP) was used to select only those **X** block features whose variations were correlated with the variations in the **Y** block. The VIP is calculated for each feature, **x**, by summing the squares of PLS loading weights, w_a_ for a = 1, 2,…,A, weighted by the amount of sum of squares explained in each model component, a.^30^ VIP values larger than 1 indicate that the feature is significant for explaining the variations in the glycosylation profile and titer, VIP values below 0.5 indicate that the feature is not significant and VIP values between 0.5 and 1 have no immediate classification as good or bad. Consequently, any feature whose VIP value exceeds 1, or whose confidence interval falls entirely above 0.5, was retained for further model building. In doing so 47 additional features were eliminated. This left 32 features that were most suitable for explaining the glycosylation profile variations: growth rate (Day 5), IVCD_24_ (Days 6 and 7), specific glutamine uptake rate (Days 0–5), specific glucose uptake rate (Days 1–3 and 6), specific lactate secretion rates (Days 5 and 6), specific glutamine uptake_24_ (Days 1–6), cumulative specific glutamine uptake (Days 1–3), specific glucose uptake_24_ (Days 3–6), cumulative specific glucose uptake (Days 3–6), and specific lactate production_24_ (Day 6).

## DISCLAILMER

The article reflects the views of the authors and should not be construed to represent FDA views or policies.

**NOTATION**
acetonitrile: ACN
antibody‐dependent cellular cytotoxicity: ADCC
Chinese hamster ovary: CHO
critical quality attribute: CQA
crystallizable fragment: Fc
design of experiments: DoE
dithiotheitol: DTT
dimethylformamide: DMF
endoplasmic reticulum: ER
immunoglobulin G: IgG
high mannose: HM
integrated viable cell density: IVCD
monoclonal antibody: mAb
multivariate data analysis: MVDA
*N*‐acetylglucosamine: GlcNAc
principal component analysis: PCA
partial least squares: PLS

## Supporting information


**Figure S1 Coefficients Plot**. The PLS model's coefficients, **B**, that relate the in‐process features, **X**, to the glycosylation profiles, **Y**, through the relationship **Y** = **XB**. The coefficients' confidence intervals are shown over the coefficients. Coefficients that are statistically significant are colored in blue, while coefficients that are not significant are colored in magenta.

## References

[1] Agarabi CD , Schiel JE , Lute SC , et al. Bioreactor process parameter screening utilizing a Plackett‐Burman design for a model monoclonal antibody. J Pharm Sci. 2015;104(6):1919‐1928. 25762022 10.1002/jps.24420

[2] Wacker C , Berger CN , Girard P , Meier R . Glycosylation profiles of therapeutic antibody pharmaceuticals. Eur J Pharm Biopharm. 2011;79(3):503‐507. 21745568 10.1016/j.ejpb.2011.06.010

[3] Lewis AM , Abu‐Absi NR , Borys MC , Li ZJ . The use of 'Omics technology to rationally improve industrial mammalian cell line performance. Biotechnol Bioeng. 2016;113(1):26‐38. 26059229 10.1002/bit.25673

[4] Rathore AS , Kumar Singh S , Pathak M , et al. Fermentanomics: relating quality attributes of a monoclonal antibody to cell culture process variables and raw materials using multivariate data analysis. Biotechnol Prog. 2015;31(6):1586‐1599. 26280800 10.1002/btpr.2155

[5] Rathore AS , Mittal S , Pathak M , Mahalingam V . Chemometrics application in biotech processes: assessing comparability across processes and scales. J Chem Technol Biotechnol. 2014;89(9):1311‐1316.

[6] Hmiel LK , Brorson KA , Boyne MT . Post‐translational structural modifications of immunoglobulin G and their effect on biological activity. Anal Bioanal Chem. 2015;407(1):79‐94. 25200070 10.1007/s00216-014-8108-x

[7] Jefferis R . Antibody therapeutics: isotype and glycoform selection. Expert Opin Biol Ther. 2007;7(9):1401‐1413. 17727329 10.1517/14712598.7.9.1401

[8] Liu L . Antibody glycosylation and its impact on the pharmacokinetics and pharmacodynamics of monoclonal antibodies and fc‐fusion proteins. J Pharm Sci. 2015;104(6):1866‐1884. 25872915 10.1002/jps.24444

[9] Fan Y , Jimenez Del Val I , Muller C , et al. A multi‐pronged investigation into the effect of glucose starvation and culture duration on fed‐batch CHO cell culture. Biotechnol Bioeng. 2015;112(10):2172‐2184. 25899530 10.1002/bit.25620

[10] Villacres C , Tayi VS , Lattova E , Perreault H , Butler M . Low glucose depletes glycan precursors, reduces site occupancy and galactosylation of a monoclonal antibody in CHO cell culture. Biotechnol J. 2015;10(7):1051‐1066. 26058832 10.1002/biot.201400662

[11] Ferrara C , Grau S , Jager C , et al. Unique carbohydrate‐carbohydrate interactions are required for high affinity binding between FcgammaRIII and antibodies lacking core fucose. Proc Natl Acad Sci U S A. 2011;108(31):12669‐12674. 21768335 10.1073/pnas.1108455108 PMC3150898

[12] Read EK , Park JT , Brorson KA . Industry and regulatory experience of the glycosylation of monoclonal antibodies. Biotechnol Appl Biochem. 2011;58(4):213‐219. 21838794 10.1002/bab.35

[13] Cymer F , Beck H , Rohde A , Reusch D . Therapeutic monoclonal antibody N‐glycosylation––structure, function and therapeutic potential. Biologicals: J Int Ass Biol Standard. 2017;52:1‐11. 10.1016/j.biologicals.2017.11.001 29239840

[14] Kildegaard HF , Fan Y , Sen JW , Larsen B , Andersen MR . Glycoprofiling effects of media additives on IgG produced by CHO cells in fed‐batch bioreactors. Biotechnol Bioeng. 2016;113(2):359‐366. 26222761 10.1002/bit.25715

[15] Grainger RK , James DC . CHO cell line specific prediction and control of recombinant monoclonal antibody N‐glycosylation. Biotechnol Bioeng. 2013;110(11):2970‐2983. 23737295 10.1002/bit.24959

[16] Seo JS , Min BS , Kim YJ , et al. Effect of glucose feeding on the glycosylation quality of antibody produced by a human cell line, F2N78, in fed‐batch culture. Appl Microbiol Biotechnol. 2014;98(8):3509‐3515. 24384750 10.1007/s00253-013-5462-0

[17] Hari SB , Lau H , Razinkov VI , Chen S , Latypov RF . Acid‐induced aggregation of human monoclonal IgG1 and IgG2: molecular mechanism and the effect of solution composition. Biochemistry. 2010;49(43):9328‐9338. 20843079 10.1021/bi100841u

[18] Liu L , Stadheim A , Hamuro L , et al. Pharmacokinetics of IgG1 monoclonal antibodies produced in humanized Pichia pastoris with specific glycoforms: a comparative study with CHO produced materials. Biologicals: J Int Ass Biol Standard. 2011;39(4):205‐210. 10.1016/j.biologicals.2011.06.002 21723741

[19] Zheng K , Bantog C , Bayer R . The impact of glycosylation on monoclonal antibody conformation and stability. MAbs. 2011;3(6):568‐576. 22123061 10.4161/mabs.3.6.17922 PMC3242843

[20] Velugula‐Yellela SR , Williams A , Trunfio N , et al. Impact of media and antifoam selection on monoclonal antibody production and quality using a high throughput micro‐bioreactor system. Biotechnol Prog. 2018;34(1):262‐270. 29086492 10.1002/btpr.2575 PMC5821576

[21] Wold S , Esbensen K , Geladi P . Principal component analysis. Chemom Intel Lab Syst. 1987;2(1):37‐52.

[22] Fan Y , Jimenez Del Val I , Muller C , et al. Amino acid and glucose metabolism in fed‐batch CHO cell culture affects antibody production and glycosylation. Biotechnol Bioeng. 2015;112(3):521‐535. 25220616 10.1002/bit.25450

[23] Goetze AM , Liu YD , Zhang Z , et al. High‐mannose glycans on the fc region of therapeutic IgG antibodies increase serum clearance in humans. Glycobiology. 2011;21(7):949‐959. 21421994 10.1093/glycob/cwr027

[24] Yu M , Brown D , Reed C , et al. Production, characterization, and pharmacokinetic properties of antibodies with N‐linked mannose‐5 glycans. MAbs. 2012;4(4):475‐487. 22699308 10.4161/mabs.20737 PMC3499342

[25] Blondeel EJM , Aucoin MG . Supplementing glycosylation: a review of applying nucleotide‐sugar precursors to growth medium to affect therapeutic recombinant protein glycoform distributions. Biotechnol Adv. 2018;36(5):1505‐1523. 29913209 10.1016/j.biotechadv.2018.06.008

[26] Aghamohseni H , Ohadi K , Spearman M , et al. Effects of nutrient levels and average culture pH on the glycosylation pattern of camelid‐humanized monoclonal antibody. J Biotechnol. 2014;186:98‐109. 25014402 10.1016/j.jbiotec.2014.05.024

[27] Swamy M , Pathak S , Grzes KM , et al. Glucose and glutamine fuel protein O‐GlcNAcylation to control T cell self‐renewal and malignancy. Nat Immunol. 2016;17(6):712‐720. 27111141 10.1038/ni.3439 PMC4900450

[28] Velugula‐Yellela SR , Kohnhorst C , Powers DN , et al. Use of high‐throughput automated microbioreactor system for production of model IgG1 in CHO cells. J Visual Exp: JoVE. 2018;(139). 10.3791/58231. PMC6235343 30320757

[29] Velugula‐Yellela SR , Powers DN , Angart P , et al. Purification and analytics of a monoclonal antibody from CHO cells using an automated microbioreactor system. J Visual Exp: JoVE. 2019;(147). 10.3791/58947. 31107445

[30] D'Errico J. SLM . Shape language modeling. *MATLAB Central File Exchange* 2018 https://www.mathworks.com/matlabcentral/fileexchange/24443-slm-shape-language-modeling. Accessed March 12, 2018.

[31] Kohavi R. . A study of cross‐validation and bootstrap for accuracy estimation and model selection. Proceedings of the 14th International joint conference on artificial intelligence; 1995; Vol 2, 1137‐1143.

[32] Chapra S . Applied numerical methods with MATLAB for engineers and scientists. 2nd ed McGraw‐Hill Science/Engineering/Math: New York, NY; 2008.

