# Supplementary figures and images for: Multivariate data analysis of growth medium trends affecting antibody glycosylation
Source: Biotechnol Prog. 2019 Oct 18;36(1):e2903. doi: 10.1002/btpr.2903 (PMC7027499; doi:10.1002/btpr.2903)

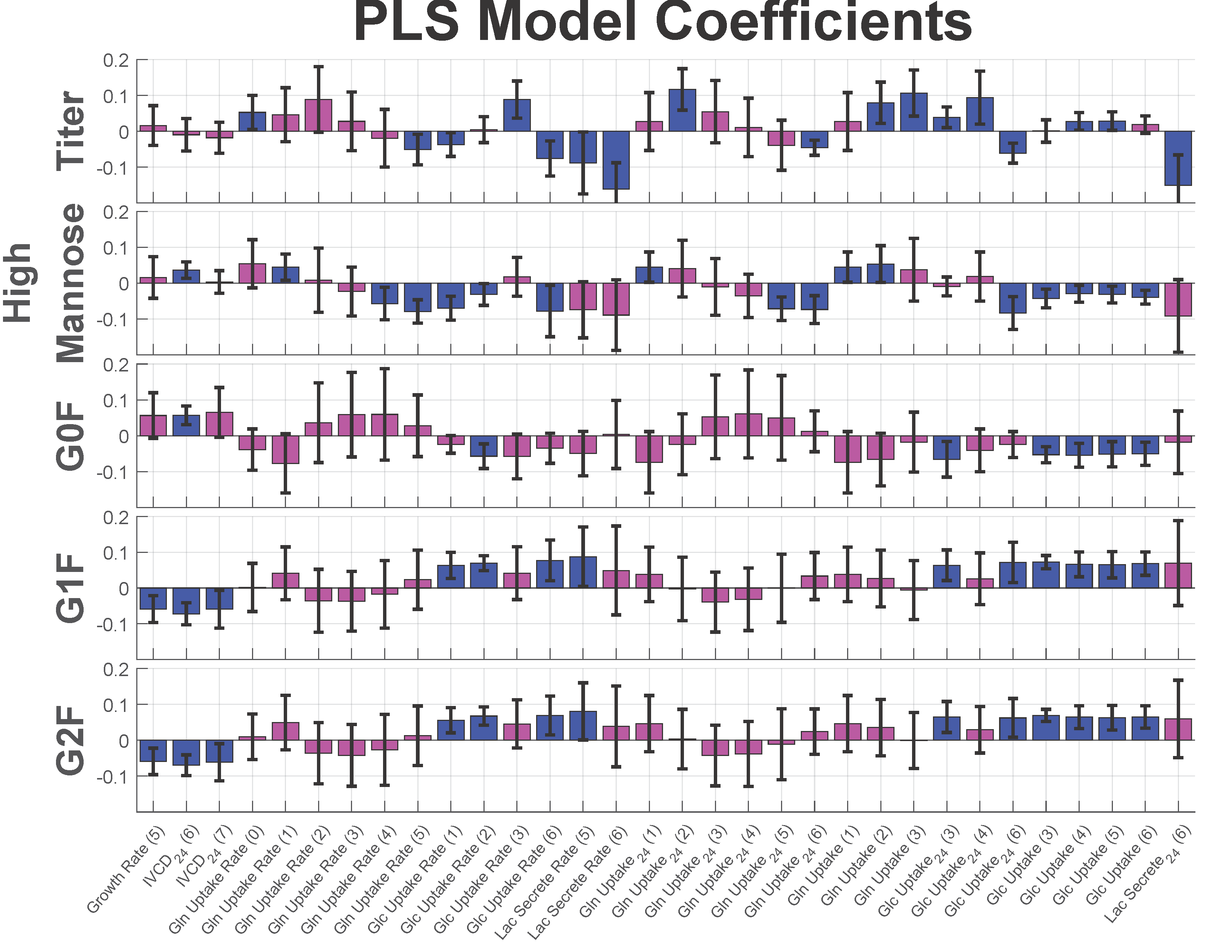

Supplement: Supplementary file 1 — Figure S1 Coefficients Plot. The PLS model's coefficients, B, that relate the in‐process features, X, to the glycosylation profiles, Y, through the relationship Y = XB. The coefficients' confidence intervals are shown over the coefficients. Coefficients that are statistically significant are colored in blue, while coefficients that are not significant are colored in magenta. [file BTPR-36-e2903-s001.tiff]
